# Mechanisms of secretion and spreading of pathological tau protein

**DOI:** 10.1007/s00018-019-03349-1

**Published:** 2019-10-30

**Authors:** Cecilia A. Brunello, Maria Merezhko, Riikka-Liisa Uronen, Henri J. Huttunen

**Affiliations:** Neuroscience Center, HiLIFE, University of Helsinki, P.O. Box 63, Haartmaninkatu 8, 00014 Helsinki, Finland

**Keywords:** Amyloid, Tau, Aggregation, Propagation, Prion, Unconventional protein secretion, Extracellular vesicles

## Abstract

Accumulation of misfolded and aggregated forms of tau protein in the brain is a neuropathological hallmark of tauopathies, such as Alzheimer’s disease and frontotemporal lobar degeneration. Tau aggregates have the ability to transfer from one cell to another and to induce templated misfolding and aggregation of healthy tau molecules in previously healthy cells, thereby propagating tau pathology across different brain areas in a prion-like manner. The molecular mechanisms involved in cell-to-cell transfer of tau aggregates are diverse, not mutually exclusive and only partially understood. Intracellular accumulation of misfolded tau induces several mechanisms that aim to reduce the cellular burden of aggregated proteins and also promote secretion of tau aggregates. However, tau may also be released from cells physiologically unrelated to protein aggregation. Tau secretion involves multiple vesicular and non-vesicle-mediated pathways, including secretion directly through the plasma membrane. Consequently, extracellular tau can be found in various forms, both as a free protein and in vesicles, such as exosomes and ectosomes. Once in the extracellular space, tau aggregates can be internalized by neighboring cells, both neurons and glial cells, via endocytic, pinocytic and phagocytic mechanisms. Importantly, accumulating evidence suggests that prion-like propagation of misfolding protein pathology could provide a general mechanism for disease progression in tauopathies and other related neurodegenerative diseases. Here, we review the recent literature on cellular mechanisms involved in cell-to-cell transfer of tau, with a particular focus in tau secretion.

## Introduction

Demographic aging is increasing the prevalence and societal cost of dementia. The number of people living with dementia is expected to reach 130 million people by 2050 (World Alzheimer Report 2015). Most chronic neurodegenerative diseases are characterized by progressive accumulation of protein aggregates in the nervous system. A common neuropathological hallmark in Alzheimer’s disease (AD), the most common type of aging-related dementia, is cerebral accumulation of neurofibrillary tangles (NFT), composed of aggregated tau protein, which appear to spread from one affected brain region to other areas of the brain as the disease progresses. Similar accumulation of tau inclusions in brain is observed in, e.g., frontotemporal dementia (FTD), progressive supranuclear palsy (PSP), corticobasal degeneration (CBD) and Pick’s disease (PiD). Slow spreading of pathologically misfolded tau protein may also be a central event in development and progression of chronic traumatic encephalopathy (CTE), which is often associated with repeated head injuries. The cellular mechanisms of spreading of pathological proteins, such as tau, α-synuclein, β-amyloid peptide (Aβ) and TDP-43, have been receiving increasing attention, and better understanding of the spreading mechanisms is expected to accelerate development of disease-modifying therapies for these devastating neurodegenerative diseases.

### Tau is a microtubule-binding protein enriched in neurons

Tau is one of the many microtubule-associated proteins (MAP) that have an important function of regulating microtubules (MT) to ensure proper cytoskeletal organization and trafficking [1], which is particularly important in highly polarized neuronal cells whose functionality and viability depend on transport of cellular cargo to and from axonal and dendritic peripheries [2–4]. Tau has important physiological functions in regulating MT, including MT polymerization, stabilization and suppression of MT dynamics. Dysregulation of the tau–MT complex leads to tau detachment and instability and disassembly of MTs, eventually leading to perturbation of MT-dependent transport and impaired maintenance of cellular polarity and viability [4, 5].

Tau is present in neurons and, to a lesser degree, in glial cells [6, 7]. Tau is mainly an axonal protein in mature neurons, but it can also be found in the nucleus, mitochondria, dendrites, synapses, and at the plasma membrane (PM) [8–13]. This localization pattern suggests that besides the main function of MT regulation, tau may have other roles in cells.

Synaptic tau has been reported to be involved in synaptic development of newborn hippocampal neurons [14]. Also, a role for tau in neuronal activity has been proposed, as tau knockout mice display impaired long-term depression in the hippocampus [11]. Tau also interacts with nucleic acids and can localize to the nucleus, and upon binding to DNA it promotes DNA stability [15], suggesting that tau may have a role in DNA protection [16]. Finally, several domains of the tau protein have the ability to interact with lipids and membranes [17], and one of tau kinases, Fyn, is mainly located in PM microdomains called lipid rafts, where tau can also be recruited [18]. It is also possible that tau localized at the PM occupies a role as a signaling regulator, via its interaction with membrane receptors [19, 20].

**Fig. 1:**
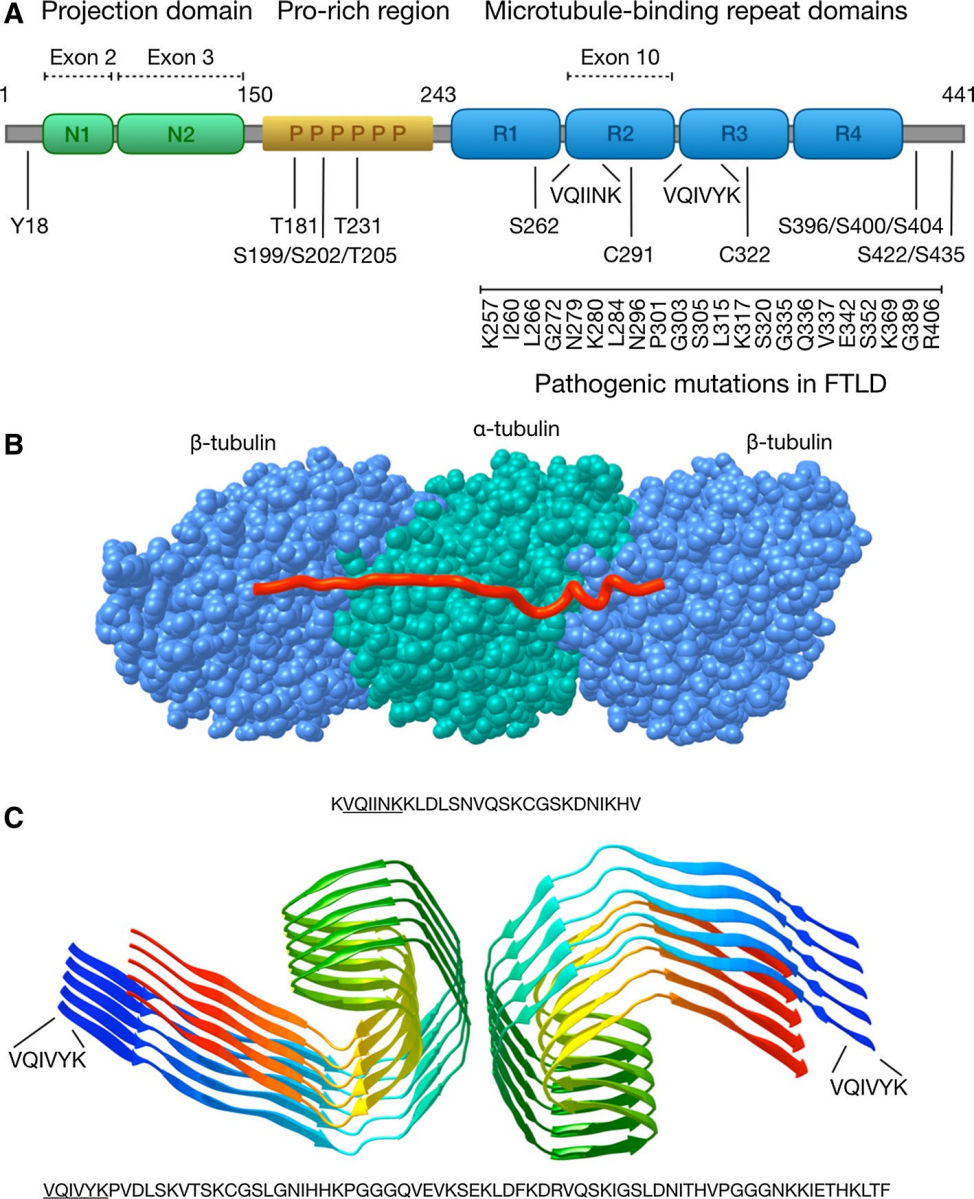
The structural basis of tau function and aggregation. **a** The domain structure of human tau protein. Location of the projection domain, proline-rich domain, MTBDs, and the parts of tau protein encoded by the alternatively spliced exons 2, 3 and 10 are shown on top of the longest tau isoform (2N4R, 441 aa). Below the location of key phosphorylated residues, the two hexapeptides, the two cysteines and examples of FTLD-associated mutations in the MTBDs are shown. **b** Microtubules (blue and green) are formed by the assembly of α- and β-tubulin dimers into protofilaments that associate laterally into hollow tubes. Tau (red) binds to the surface of microtubules interacting with α- and β-tubulin via the MTBDs. The image was prepared based on a cryo-EM structure of microtubule-associated synthetic tau (PDB: 6CVN). **c** Structure of a paired helical filament (PHF) fragment isolated from AD brain. The filaments are formed of anti-parallel β-sheets, with the protofilament core formed by the four MTBDs of tau. The location of the tau hexapeptide sequence is indicated. Image was prepared based on a cryo-EM structure (PDB: 5O3L)

Tau is encoded by the *MAPT* gene, which in humans is located on the chromosome 17. Tau is composed of 16 exons which give rise to six different splicing isoforms, which span from 352 to 441 amino acids (aa) [21], depending on alternative splicing of exons 2, 3 and 10 (Fig. 1a). Exons 2 and 3 encode for 29 amino acid repeats, both located at the N-terminal part of the protein, and alternative splicing of exons 2 and 3 or exon 2 alone produces three N-terminal protein variants 0N, 1N (additional 29 aa) or the less abundant 2N (additional 58 aa). Exon 10 encodes for one of the four possible microtubule-binding repeat domains (MTBD), which are 31–32 aa long imperfect repeat sequences, located in the C-terminal half of the tau protein and affecting both microtubule-binding affinity and fibrillization properties of tau. Splicing isoforms of tau contain either three (3R) or four (4R) MTBDs, which affects their microtubule-binding affinity (4R > 3R) and also their propensity for aggregation. The ratio between 3R and 4R isoforms is developmentally regulated as human fetal tau consists mostly the shortest form 0N3R, while in the adult brain all six isoforms coexist. Also, species-specific differences exist, as for example adult mice display exclusively 4R-tau isoforms [22].

Different functional domains characterize the tau protein (Fig. 1a). The N-terminal projection domain consists of residues 1–150 (of the longest isoform) and it regulates microtubule binding even though it is not directly involved in the physical interaction [23]. Absence of the N-terminus alters cellular localization of tau, promoting cytosol to nucleus relocalization [24]. It has also been suggested that the N-terminal projection domain mediates PM localization of tau via annexin-2 interaction [25]. The proline-rich domain occupies the central portion of tau from residue 151–243. It represents the most disordered part of the protein and serves as an interaction site for Src homology-3 (SH3) proteins, in particular the Fyn kinase [26], as well as an interaction site for DNA and RNA [27, 28]. The MTBDs, as the name suggests, interact with microtubules but also with actin, orchestrating the order and stability of the cytoskeleton. Several proteins associated with neurodegenerative disorders, including α-synuclein, presenilin-1, FUS, and TIA-1, interact with the MTBDs and the proline-rich domains of tau, suggesting that the structural composition and protein–protein interactions of tau could play an important role in pathological processes [29].

Mutations in the *MAPT* gene have been linked to hereditary dominant frontotemporal dementia with parkinsonism in chromosome 17 (FTDP-17), which includes diverse clinical syndromes as well as diverse anatomical distribution of tau inclusions depending on the specific mutations [30]. First mutations in the MAPT gene were found in 1998 [31, 32] linked to dominant hereditary FTDP-17, and today there are over 50 known pathogenic mutations in the MAPT gene, mostly located in exons 9–12 [30]. Such mutations can affect tau both at the protein level, for example by altering microtubule-binding properties (e.g., K257T, G272V, P301L, V337M and R406W mutations), and at the RNA level, shifting the splicing toward overproduction of 4R-tau (e.g., N279K, N296H, P301S, S305I mutations) [33, 34]. This is relevant because isoforms containing exon 2 and exon 10 are more aggregation prone compared to isoforms containing only exon 3, due to the presence of the VQIINK hexapeptide (encoded by exon 10) that mediates homo-oligomerization [35, 36]. Additionally, alterations in the ratio of physiologically produced 3R-tau and 4R-tau may cause tau dysfunction in cells, even in the absence of mutations affecting splicing of exon 10. For instance, 4R-tau binds to microtubules with higher affinity compared to the 3R isoforms, promoting microtubule assembly and oversaturation of the available binding sites [37]. In turn, an imbalance between 4R-tau and 3R-tau isoforms may cause accumulation of unbound 4R-tau in the cytosol, which would favor tau aggregation.

### Structural plasticity and aggregation of tau

Although tau is considered a natively unfolded protein [sometimes also called an intrinsically disordered protein (IDP)], its ability to adopt various structural states is important to its functions and pathological properties. Since the first report on tau structure in 1977 [38], there have been numerous approaches and reports addressing tau structure in various contexts (reviewed in [39]). While the intrinsically disordered nature of tau precludes the use of crystallographic methods, circular dichroism, nuclear magnetic resonance (NMR) spectroscopy, and more recently cryogenic electron microscopy (cryo-EM) have provided insight into conformational states of tau, in soluble physiological state, bound to microtubules or in the form of pathological aggregated states. It was also recently suggested that under physiological conditions tau monomers may adopt stable yet diverse conformations, opening a new perspective for understanding the plasticity of tau structure in physiology and in pathology [40, 41].

Multiple tau monomers bind in tandem to the surface of microtubule (MT) protofilaments. The conserved MTBDs adopt similar extended structures along the microtubules, stabilizing the interface between tubulin dimers [42] (Fig. 1b), while the N-terminal projection domain extends away from the MT protofilament surface. While microtubule binding does not promote adoption of distinct secondary structures, the MTBDs may have some α-helical propensity. Also, interaction with membranes may facilitate adoption of temporary secondary structures.

The ability of tau to aggregate is encoded in its own sequence. Especially, two hexapeptides present in the MTBDs (VQIVYK and VQIINK) are fundamental for aggregation and formation of the tau filaments [36, 43]. One of the hexapeptides (VQIVYK) is present in all tau isoforms in the third MTBD, while the other one (VQIINK), since it is located in the second MTBD encoded by exon 10, is only present in the 4R isoforms. This partially explains the higher propensity of 4R isoforms to aggregate compared to 3R isoforms. Local structural changes of the MTBDs via exposure of the VQIVYK/VQIINK motifs may determine the conversion of an inert tau monomer into a pathologically aggregated form [40]. Tau dimerization, the first step in tau oligomerization, can occur through any combination of these two motifs, which form the nucleation center where further tau dimers and tau monomers can be recruited to eventually constitute oligomers. Of particular importance are also two cysteine residues located in R2 and R3, which form disulfide bridges during tau dimerization and which have been found to constitute a critical step in the formation of tau fibrils [44]. Elongated oligomers then acquire a more ordered β-sheet structure, and finally form paired helical filaments (PHFs), which represent the building blocks of the neurofibrillary tangles (NFT) [45]. Cryo-EM structures of tau filaments extracted from AD brain confirmed the β-sheet-dominant pathological conformation [46], confirming previous findings [47]. Straight and paired helical filaments appear to be ultrasctructural polymorphs, both sharing the same basic structural architecture (Fig. 1c).

The widely studied P301L mutation, found in some patients with FTDP-17, occurs near one of the hexapeptides and promotes tau aggregation [31]. On the other hand, artificial mutations in the hexapeptides, such as proline insertion that distort the β-sheet structure, reduce tau aggregation [48]. In addition, the cellular environment, such as the presence of anionic membranes, also promotes tau aggregation [49, 50]. A similar effect results also from polyanions such as heparin and RNA [51, 52].

Among the many post-translational modifications that modulate tau functions, phosphorylation is of particular interest because of its role in the pathological processes, as the deposits of insoluble tau found in the brains of tauopathy patients contain hyperphosphorylated tau [53–56]. Since the first report that classified tau as a phospho-protein [38], there are now at least 85 known phosphorylation sites (mostly serines and threonines, but also tyrosines). Tau is physiologically constantly phosphorylated and dephosphorylated to ensure regulation of proper functions, but when the balance is shifted toward phosphorylation, tau affinity for microtubules decreases [57]. This results in an increase in cytosolic tau, which becomes more vulnerable to aggregation [58]. Additionally, abnormally phosphorylated tau is able to sequester other microtubule-associated proteins (MAP), further worsening microtubule destabilization [57]. It is now well established that there is a strong connection between aberrant phosphorylation and self-aggregation of tau into oligomers and higher-order aggregates [36], despite the fact that a complete pathological phosphorylation pattern has not been identified yet. The neurodegeneration-associated consequences of tau hyperphosphorylation also include impaired axonal transport [59], cellular relocalization of tau to the somatodendritic compartment and synaptic loss [60]. Synaptic dysfunction can occur both pre-synaptically, where phosphorylated tau has been shown to interfere with synaptic vesicles [61], and post-synaptically via downregulation of AMPA receptors [62]. Interestingly, it was shown that synaptic terminals of AD brains and transgenic P301L mice do not contain more tau compared to control brains, indicating that there is no relocalization to synapses in pathological conditions. Instead, synaptic tau was hyperphosphorylated, suggesting a role for phosphorylation in the spreading of the disease as well [63, 64]. Additionally, hyperphosphorylation is linked to impaired degradation of tau by the ubiquitin–proteasome system [65] and to tau secretion. It was in fact shown that abnormally phosphorylated tau is secreted more efficiently than non-phosphorylated tau, at least in cell lines [66, 67]. Finally, abnormal tau phosphorylation impairs its ability to interact with tau partners, therefore altering normal physiological properties of tau [68].

Tau phosphorylation state is a result of a tightly regulated balance of actions between many cellular proteins: mainly kinases that phosphorylate tau and phosphatases that dephosphorylate it. Tau kinases are divided into three different groups: (1) serine/threonine proline-directed kinases, which include GSK3, CdK5, MAPKs and other stress-activated kinases; (2) serine/threonine non-proline-directed kinases, which include DYRK1A, PKA, CaMKII and CK1; and (3) tyrosine kinases, such as Fyn and Src [69]. Some kinases, such as GSK3β, MAPK, CK1δ and Cdk5, are found in neurofibrillary tangles, suggesting a direct link between phosphorylation and disease progression [55]. The activity of phosphatases like PP2A, one of the principal neuronal protein phosphatases, is dramatically decreased in the brain of AD patients [70]. This suggests that not only upregulated kinase activity, but also downregulated phosphatase activity is involved in pathological phosphorylation of tau. There likely exist complex regulatory loops that maintain tau phosphorylation in homeostasis. For instance, impairment of the Akt/mTOR pathway may alter the physiologic phosphorylation balance between GSK3β and PP2A, as Akt inhibits GSK3β, which in turn inhibits PP2A [22].

Our understanding of the intricate mechanism of tau phosphorylation equilibrium is further complicated by an increasing amount of evidence that challenge the classical view that correlates hyperphosphorylation with neurodegeneration [71]. For instance, phosphorylation sites that promote tau disassembly from the microtubules not only favor tau aggregation, but can actually inhibit it [72]. In line with these results, Arendt and colleagues suggested that hyperphosphorylation of tau may have a physiological role, possibly related to synaptic plasticity [73].

These observations lead to a very important conclusion, which was suspected for a long time but only recently experimentally proven: considering all the factors that affect tau aggregation (mutations, isoform composition, post-translational modifications and localization), it seems unlikely that a single form of tau is responsible for its pathological effects. It is more likely that a plethora of different pathological tau species with different properties accounts for the heterogeneity observed in tau-mediated pathology [74]. Moreover, tau pathology is rarely exclusively present in any of the tauopathies, as other pathological proteins and other phenotypes often accompany tau pathology and dysfunction. There may therefore be some common pathways that bring together different proteins with very different cellular functions, possibly causally linking development of distinct pathological features and resulting in synergistic modes of toxicity. For example, in mouse model of AD, soluble Aβ promotes development of tau pathology [75, 76]. In tau, presenilin1 and APP transgenic mice, amyloid pathology precedes development of NFT pathology [77]. Tau and α-synuclein promote fibrillization of each other [78] and insoluble forms of tau and α-synuclein have been found co-aggregating in Lewy bodies, the characteristic pathological deposits of Parkinson’s disease (PD) [79]. Moreover, genetic studies have linked variants of the tau gene, MAPT, to susceptibility of developing sporadic PD [80, 81], and mutations in MAPT are associated with dementia with parkinsonism [31]. It is therefore clear that the neuropathological features that characterize different tauopathies could originate from different types of tau modifications and interactions with other cellular proteins, defining different strains of pathological tau with different molecular properties.

### Tau inclusions in neurodegenerative diseases

Tau dysfunction can lead to a wide variety of human disorders, called tauopathies, which include very different clinical syndromes and a constellation of pathological hallmarks. The principal tauopathies include AD, FTDP-17, the spectrum corticobasal degeneration/progressive supranuclear palsy (CBD/PSP), Pick’s disease and argyrophilic grain disease (AGD). A list of the main tauopathies, with their key features, is presented in Table 1.

**Table 1:** The principal neurodegenerative disorders characterized by tau dysfunction and inclusions

Disease	Familiar/sporadic	Main brain areas affected	Clinical symptoms	Neuronal tau inclusions	Other inclusions	References
AD	F/S	Entorhinal region, hippocampus, occipital, temporal and parietal cortex	Dementia with learning, language, reasoning, orientation deficits. Behavioral alterations	3R/4R tau. NF tangles with PHF, neuropil threads, neuritic plaques	Aβ inclusions, TDP-43 inclusions, Hirano bodies, granulovacuolar degeneration. Tau glial inclusions	[239]
FTDP-17	F	Highly variable depending on mutations. Frontal and temporal gyri, anterior temporal lobe	Behavioral and personal abnormalities, cognitive deficits with aphasia and parkinsonism	3R/4R tau. NF tangles and Pick’s bodies. Filament morphology depends on MAPT mutations	Tau coiled bodies in oligodendroglia, tufted astrocytes, astrocytic plaques	[30]
PSP CBD	S	Highly variable depending on sub-syndromes. Mild atrophy in posterior frontal and precentral gyri (PSP), and superior frontal and parietal regions (CBD)	Variable depending on sub-syndromes. Dementia, rigidity apraxia, non-fluent aphasia, parkinsonism	4R-tau. Flame-shape NF tangles and globose NF tangles (corticobasal bodies in monoaminergic neurons). Neuropil threads	Tufted astrocytes in PSP, astrocytic plaque in CBD, coiled bodies in oligodendroglia in both	[240, 241]
PiD	F/S	Atrophy in frontal, temporal and sometimes parietal lobe, and limbic structures	Speech impairment, aphasia, disinhibition, apathy	3R-tau. Pick’s bodies, few NF tangles	Sparse tau glial inclusions	[242]
AGD	S	Mostly unchanged, mild atrophy in frontotemporal cortex	Mild cognitive impairment	4R-tau. Argyrophilic grains, NF tangles in limbic areas. Straight filaments	Coiled bodies in oligodendroglia	[82]
Parkinsonism-dementia complex of Guam	S	Diffused atrophy, particularly in frontotemporal lobes, hippocampus, parahippocampus and white matter	Rigidity, tremors, bradykinesia, dementia, olfactory dysfunctions	4R-tau. NF tangles with PHF and straight filaments. Neuropil threads	Astrocytic hazy inclusions, coiled bodies in oligodendroglia	[243]

*AD* Alzheimer’s disease, *FTDP-17* frontotemporal dementia and parkinsonisms linked to chromosome 17, *PSP* progressive supranuclear palsy, *CBD* corticobasal degeneration, *PiD* Pick’s disease, *AGD* argyrophilic grain disease

Tauopathies are typically associated with dementia and motor impairments, but the clinical picture can vary between different disorders and even within syndromes, ranging from minor cognitive impairments [82] to the severe behavioral changes and cognitive and motor impairments of FTDP-17 [83]. Similarly, while it is quite common for the majority of tauopathies to be characterized by generalized cortical atrophy and ventricle enlargement, which partially explains the overlapping symptoms, the progression pattern and affected brain areas may vary [84]. While biochemical, genetic, pathological and clinical similarities between tauopathies can facilitate understanding of the general principles of how tau pathologies develop, they also complicate the diagnosis and classification of different tauopathies.

Tauopathies can be classified as primary or secondary, depending whether tau inclusions constitute the main pathological hallmark or whether other pathological proteins significantly contribute to the pathogenesis of the disease [84]. For instance, AD, the most common neurodegenerative disease and one of the main models for the investigation of tau pathology, is a secondary tauopathy to amyloid plaques, which are largely composed of aggregated Aβ peptides [85]. Primary tauopathies include FTDP-17, CBD/PSP, PiD and AGD. Majority of tauopathies are sporadic, but for example FTDP-17 is strictly inherited and all the different clinical and neuropathological subtypes of FTDP-17 are caused by different mutations in the MAPT gene [86]. Notably, regarding the classification of nervous system diseases, it was recently proposed that the term FTDP-17 should be retired and FTDP-17 cases should be considered as familial forms of frontotemporal lobar degeneration with tau-immunoreactive inclusions (FTLD-tau) [87].

Classification of tauopathies can also be done based on the tau isoforms present in the inclusions and morphology of the tau filaments found in the pathological deposits. While AD and FTDP-17 are mixed 3R and 4R tauopathies, reflecting the physiological ratio of expressed tau isoforms in the healthy brain [88], CBD/PSP, and AGD are classified as 4R tauopathies, as the pathological inclusions are predominantly constituted by 4R-tau [84]. The only known tauopathy characterized by inclusions of 3R-tau is Pick’s disease [89]. Tau inclusions also differ regarding the morphology of tau filaments and the cell types where they are deposited. The evidence that different missense mutations in the MAPT gene can lead to different filament morphology gives an idea of the possible morphological heterogeneity of tau aggregates. For instance, N279K mutation promotes formation of tau bundles of varying thickness [90], while P301L mutation causes accumulation of twisted ribbons and stranded filaments [32] and S305I mutation promotes formation of straight and tubular filaments [91].

Specific tauopathies may display specific types of tau inclusions, as shown by argyrophilic grains and Pick’s bodies. Pick’s bodies are round and well-demarcated structures, normally occurring one per cell and constituted by heterogeneous types of tau filaments [92]. Unlike Pick’s bodies, argyrophilic grains result positive for Gallyas staining and are characterized by a spindle shape mostly composed of straight tau filaments [93]. CBD/PSP and FTDP-17 on the other hand display pleomorphic neuronal tangle-type inclusions, which vary in size, shape and distribution [94–96]. Moreover, in the majority of tauopathies glial inclusions, in the form of coiled bodies, are found in oligodendrocytes [97, 98], astrocytic tufts and astrocytic plaques [99].

A recurring theme in neurodegenerative diseases is the co-occurrence of protein inclusions composed of different proteins in the same patient [68, 100]. For example, tau and α-synuclein inclusions co-occur in multiple diseases including Lewy body variant of AD, DLB and PD with dementia [101, 102]. Despite the fact that tau and α-synuclein are distinct proteins that contribute to different disease-specific pathologies, multiple lines of evidence suggest that tau and α-synuclein interact, modulate the aggregation of each other and coexist in pathological inclusions in human brain [103]. Apart from the potential gain-of-toxic-function relationship that feeds forward the aggregation of each other, tau and α-synuclein may also overlap in their loss-of-function effects via a partially shared protein interactome [68]. Similar cross talk may occur between other disease-associated aggregating proteins, e.g., between tau and TDP-43 [104].

In AD, autopsy studies have demonstrated that pathological Aβ and tau inclusions accumulate in stereotypical spatial patterns over the course of the disease with the AD-related tauopathy occurring downstream of amyloid pathology [105]. Development of PET tracers specifically binding to the aggregated forms of Aβ and tau now allows a better understanding of the temporal relationship of the amyloid and neurofibrillary pathologies in the human brain. It seems that the level of tau deposition in the temporal lobe is more closely related to dementia status and can predict cognitive performance better than Aβ deposition in any brain region [106].

### Spreading of tau pathology in brain

It is well established that in AD the development of tau pathology occurs in a hierarchical pattern of accumulation starting from the layer II of entorhinal cortex (EC) and spreading toward cortical regions, as originally proposed by the findings of Heiko and Eva Braak [107]. Increased cerebrospinal fluid (CSF) levels of tau are associated with faster rate of cognitive decline and overall worse clinical outcome in AD [108, 109]. Accumulating evidence suggests that in most neurodegenerative diseases, inclusions of misfolded and pathological proteins spread along neuroanatomically connected areas of the brain in a way that remind the spread of pathological prion protein (PrP) [110]. It was previously thought that stage-wise progression of neurodegeneration would reflect a differential spatial and temporal vulnerability of different brain areas to protein misfolding pathology and toxicity, therefore explaining the anatomical progression of the pathological inclusions [111]. However, the concept that pathologically misfolded protein species, such as tau aggregates, can be released from a cell and internalized by a neighboring cell from the extracellular space in a prion-like manner [110] has provided a new way of approaching the progression of tauopathies and other related neurodegenerative diseases.

Tau is able to serve as a hub for its own aggregation and as a seed for further misfolding in a process called templated misfolding. Once aggregated, the pathological tau seeds can be transmitted from one cell to another propagating pathology from affected to healthy cells and to previously unaffected brain areas. In 2009, a pioneer study demonstrated that tau extracted from tau(P301S) transgenic mice, which carries one of the frontotemporal dementia tau mutations and develops tau inclusions, and injected into healthy mice that do not normally develop tau inclusions, lead to development of tau pathology [112]. The authors detected extensive distribution of phosphorylated, filamentous and insoluble tau by silver staining and antibody labeling in the areas near the injection, as well as in neighboring areas of the brain, indicating that tau pathology can be seeded and propagated. A similar pathological propagation phenomenon has been reported in a number of studies, for e.g., tau extracted from human AD brains [113, 114] and from mice exposed to traumatic brain injury (TBI) [115].

Both in vitro and in vivo studies of tau propagation have been facilitated with the use of pre-formed fibrils (PFF) of tau, originally developed in the laboratory of John Trojanowsky and Virginia Lee [116]. Experiments performed with tau PFFs, both in vitro and in vivo, have revealed that tau can propagate trans-synaptically from one neuron to another, and that the nearby network, synaptic contacts and neuronal activity modulate such propagation [117, 118], possibly bridging the physiological release of tau [119, 120] and the pathological one [121]. Recent studies, using novel PET tracers binding to PHF-tau allowing real-time visualization of tau pathology, also support the view that progression of tau pathology occurs in the brains of human AD patients [122, 123].

It should also be taken into account that despite tau being a cytosolic and microtubule-associated protein, a physiological role for secreted tau is possible. Some evidence suggests that neuronal activity can influence tau release at the synaptic terminal and that such release is not connected to propagation of tau or transfer of any type of pathology to neighboring neurons [119, 120]. Moreover, despite the origin and the degree of propagation substantially differing in different diseases, it is plausible that some common mechanistic features underlie the spread of pathology in various neurodegenerative diseases with different characteristic aggregating proteins. Thus, there is a clear need for better understanding of the specific mechanisms that drive tau secretion and of the specific forms of secreted tau and their different pathogenicity.

## Cell-to-cell transfer of tau

**Fig. 2:**
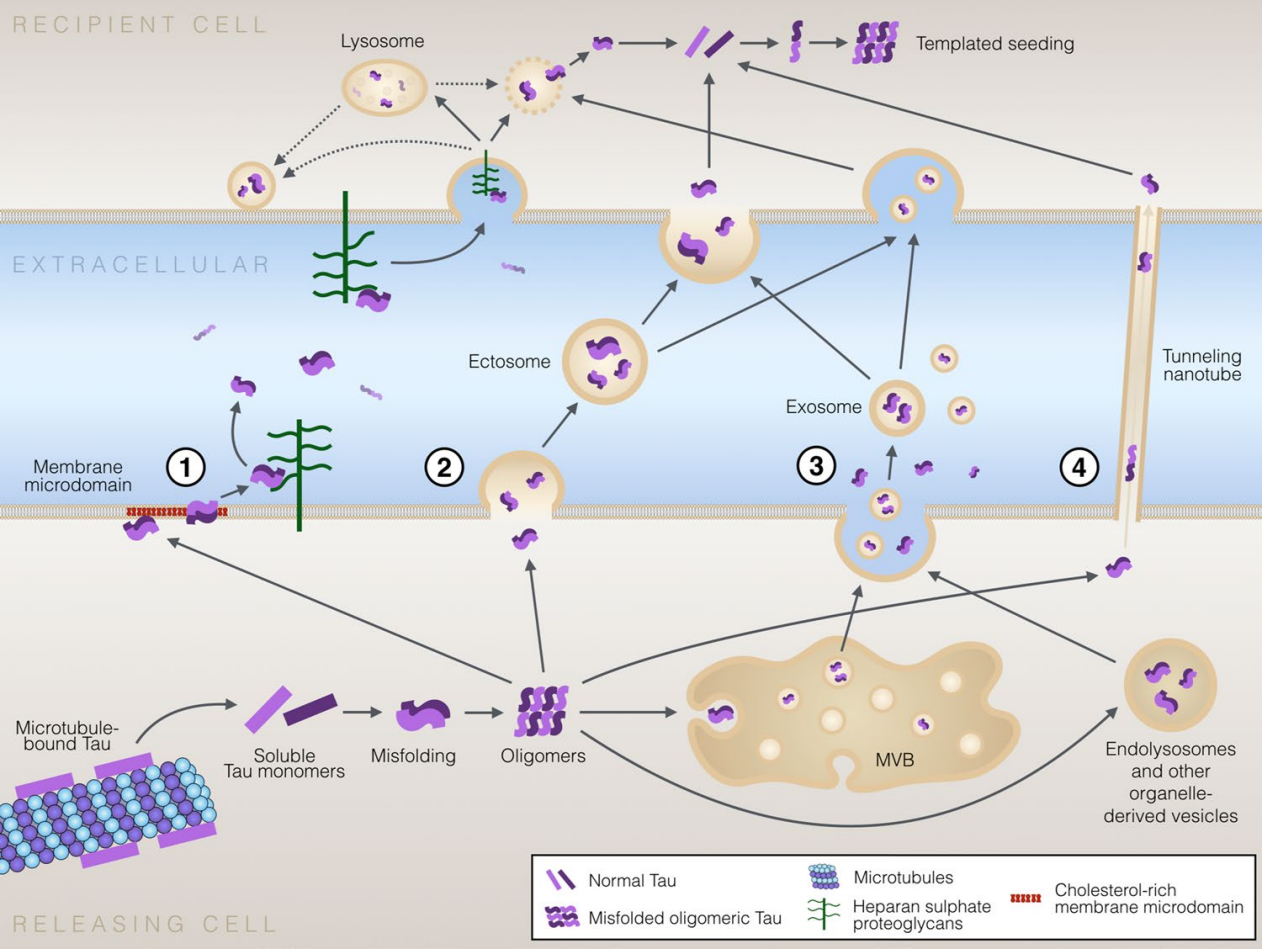
Mechanisms of cell-to-cell transfer of pathological tau protein. Pathological tau conformers can be transferred between cells by multiple non-exclusive mechanisms. (1) Tau secretion directly through the PM involves clustering of tau at the PM, interaction with specific lipids in cholesterol/sphingomyelin/PI(4,5)P_2_-rich membrane microdomains, penetration through the PM and release from the PM facilitated by cell surface HSPGs. This unconventional tau secretion mechanism resembles the secretion of FGF2 (UPS I-like). (2) Tau is secreted in ectosomes shed from the PM. Ectosomes are larger than exosomes and also differ in their molecular composition. After their release from cells, both ectosomes and exosomes function similarly and can be fused to or endocytosed by target cells. (3) Secretion of tau in exosomes and via organelle hitchhiking. Tau can be packed in exosomes by inward budding of late endosome membrane leading to formation of intraluminal vesicles in multivesicular bodies (MVB) that can be secreted by fusion of MVB membrane with the PM. Other organelle hitch-hiking (UPS III-like) pathways possibly involved in secretion of tau and other misfolded cytosolic proteins include secretory endo-lysosomes, related to the autophagy-lysosomal pathway. The MAPS pathway promotes secretion of cytosolic misfolded proteins by chaperone-mediated capture of misfolded cytosolic proteins to the ER, followed by secretion via fusion of endo-lysosomal vesicles with the PM releasing vesicle-free tau in the extracellular space. (4) Cell-to-cell transfer of tau seeds via tunneling nanotubes that directly connect the cytosols of two neighboring cell. Regardless of the secretion pathway, tau aggregates eventually reach the cytosol of the recipient cells, allowing templated seeding of healthy tau molecules into misfolded pathological conformations. The recipient cells can then propagate the pathology further to other previously unaffected cells. It is currently unclear which ones of the above mechanisms are involved in synaptic release of tau, and whether the synaptic release of physiological and pathological forms of tau are mediated by the same mechanism(s)

Despite the fact that tau is a microtubule-associated cytosolic protein, it is physiologically present outside the cells. While many aspects of tau secretion remain unknown, it seems that tau can escape from cells via multiple routes (Fig. 2). Tau is found in the CSF [124], and CSF of AD patients displays elevated levels of phosphorylated tau species that seem to correlate with increased disease severity [124, 125]. These findings suggest a prominent role for extracellular tau in disease pathogenesis and progression. Importantly, while a fraction of tau found in the cerebrospinal fluid may be associated with passive release of the intracellular content from dying cells, multiple lines of evidence suggest that active cellular processes are involved in tau secretion.

Some studies have shown that tau is secreted at the synaptic terminus during normal synaptic activity [119, 120]. This constitutive and physiological secretion of tau has not been directly linked to propagation of pathological forms of tau to neighboring neurons, although the long-term effects of physiologically secreted tau are not known yet. It is also possible that secreted monomeric tau could have some yet unknown physiological signaling activity. Other studies, however, suggest that presynaptic neuronal activity may modulate the release and trans-synaptic transfer of pathological tau [126, 127]. Furthermore, in animal models pathological tau appears to be more localized to synapses compared to non-pathological tau [63], and synaptosomes isolated from human AD brains were shown to contain more phosphorylated and aggregated tau than in healthy controls [64]. Although several studies have demonstrated that presynaptic neuronal activity enhances tau release, the mechanism of this phenomena, however, remains poorly understood. Neuronal depolarization or the associated presynaptic activity and Ca^2+^ response can modulate the release of two types of extracellular vesicles, ectosomes and exosomes [128, 129]. Indeed, Wang and colleagues have demonstrated that depolarization of neurons promotes the release of tau-containing exosomes [127]. Pooler et al., however, suggested that synaptic activity mainly modulates non-exosomal secretion of tau, through a mechanism dependent on the release of synaptic vesicles [119]. While it is still controversial how tau secretion occurs from synaptic terminals, whether associated with vesicles or via vesicle-free mechanism, it seems that in pathological conditions synapses are important for the spread of the pathogenic protein. Pathological spread of another misfolded protein, α-synuclein, was recently shown to occur via the brain connectome [130] and it is plausible that similar neuronal network-mediated spread of tau pathology happens in tauopathies. This is supported by the evidence showing that the transmission of tau pathology occurs via a trans-synaptic mechanism, and that the pattern of spread is determined by synaptic connectivity rather than spatial proximity [131].

While in vivo studies have been critical for determining the cross-species seeding abilities of different pathological tau strains [132] and in demonstrating that pathological inclusions from human brains affected with tauopathies induce NFT formation in rodents, often recapitulating the disease-specific features of the original inclusion [113, 133, 134], the molecular mechanisms underlying tau propagation remain challenging to assess in vivo. In vitro approaches have significantly contributed to our understanding of the basic mechanisms involved in the cell-to-cell transfer of tau pathology by, e.g., showing that morphologically distinct tau seeds can induce similar types of tau aggregates in recipient cells, surprisingly faithfully recapitulating morphological features of tau aggregates [74, 135]. Diverse molecular pathways that are involved in cell-to-cell transfer of pathological tau have been identified, drawing a heterogeneous landscape of mechanisms by which tau can exit cells and enter neighboring cells. Table 2 summarizes the currently available evidence supporting various cellular mechanisms involved in secretion of tau. There has been much focus on vesicular mechanisms, including exosomal secretion and release in PM-shed ectosomes, but also reports on vesicle-free secretion resulting in naked vesicle-free tau in the extracellular space. Different mechanisms are also involved in cellular uptake of vesicular tau and vesicle-free tau, and this likely affects the efficiency of cellular uptake, subcellular localization of internalized tau and the overall seeding potency of different forms of tau and in different cell types.

**Table 2:** Summary of published evidence supporting different pathways involved in tau secretion

Mechanism	System	Cellular tau			Extracellular tau			References
		Endogenous	Overexpressed WT	Overexpressed mutated	Vesicle-associated tau	Vesicle-free tau	Description of extracellular tau	
Unconventional secretion (UPS I-like, directly through the plasma membrane)	Cell line		X		0–7%	93–100%	Phosphorylated, soluble oligomers	[67, 158, 159]
		X			0%	100%	Full-length, monomeric	[244]
	Primary neurons	X			N/D	N/D		[67, 159]
	iPSC neurons	X			N/D	N/D	Phosphorylated (T231)	[245]
Secretion using intracellular vesicles (UPS III-like)	Cell line		X		< 10%^a^	< 90%^a^	Vesicular: tau fragments	[246]
			X	(X)	Yes	Yes/N/D	Phosphorylated (T181, S199, T231, S262, 361 S356, and S396); vesicular: C-term fragments, phosphorylated	[145, 154, 155, 247]
		X			Yes	Yes		[152]
		X	(X)	(X)	N/D	N/D		[149]
			X	(X)	Yes/N/D	N/D	Vesicular: full-length tau, 12% of tau is sarkosyl-insoluble aggregates	[127, 153]
	Primary neurons	X	X		Yes	N/D		[155]
		X			N/D	N/D		[149, 153, 154]
		X			2%	98%	Vesicular: full-length tau, hypophosphorylated^b^	[127]
		X			Yes	Yes	Vesicular: full-length tau and fragments	[151]
	iPSC neurons	X		X	100%	0%	Vesicular: contains phosphorylated (S396/S404) and misfolded tau	[248, 249]
		X			< 10%^a^	< 90%^a^	Vesicular: 80% of tau is full-length tau; Vesicular-free: only 17% of tau is full-length tau	[250]
	Rodent brain/ISF	X	X	(X)	Yes	N/D	Vesicular: mostly full-length tau, phosphorylated	[147, 155]
		X		(X)	Yes/N/D	N/D	Vesicular: full-length and fragments; hypophosphorylated^b^, oligomeric (only in transgenic mice)	[143, 144, 149, 251]
	Human brain/CSF	X			Yes/N/D	Yes/N/D	Vesicular: phosphorylated, monomeric and oligomeric tau fragments	[127, 145, 151]
		X			< 10%^a^	< 90%^a^	Vesicular: 6% of tau is full-length tau Vesicle-free: 1% of tau is full-length tau	[250]
	Human blood/plasma exosomes	X			Yes	N/D	Vesicular: phosphorylated (T181, S396)	[146, 252–255]
	Lamprey giant neurons		X		N/D	N/D		[256]
Ectosome/microvesicle shedding	Cell line		X		Yes	N/D	Vesicular: predominantly C-terminal fragments	[155]
	Primary neurons	X			10%	90%	Ectosomal: full-length and C- or N-terminally truncated	[155]
	Rodent brain/ISF	X			Yes	N/D	Ectosomal: full-length tau and fragments	[155]
	Human brain/CSF	X			Yes	N/D		[156]
	Lamprey giant neurons		X		N/D	N/D		[256]
Mechanism unclear	Cell line	X	(X)		no	N/D		[257]
			X		> 1 to 10%	90 to < 99%	Monomers, dimers and soluble pre-aggregates or fibrils	[157, 258]
			X		Yes	< 90%^a^	Full-length and C-terminally truncated fragments; hypophosphorylated^b^ (S202, T205, S422, S396/S404)	[66]
	Primary neurons	X	(X)	(X)	0 to > 20%^a^/N/D	< 80%^a^ to 95%/N/D	Soluble small oligomers (mostly dimers); Full-length tau, largely dephosphorylated (Ser396/404, Ser199/202/Thr205)	[119, 121, 258, 259]
	iPSC neurons	X			N/D	N/D		[121]
	Rodent brain/ISF	X		(X)	N/D	N/D		[120, 121]
	Human brain/CSF	X			N/D	N/D	Full-length and truncated forms	[126]

*X* analysis was done in a separate experiment or only in part of the experiments shown

^a^Estimation based on provided images or graphs (quantitative data not provided by the authors)

^b^Compared to cellular tau

In addition to transfer of tau between cells via the extracellular space, a direct intercellular mechanism via tunneling nanotubes allows transport of cytosolic tau directly from the cytoplasm of one cell to the cytoplasm of another cell [136] (Fig. 2). Tunneling nanotubes, up to 100-μm long communication paths between cells [137], have been shown to deliver pathological protein aggregates, including PrP, α-synuclein, tau, huntingtin and Aβ, in naked form or associated with lysosomes [138]. Interestingly, tunneling nanotubes can mediate the transfer of pathological proteins not only between two neuronal cells, but between astrocytes as well [139]. Moreover, intracellular misfolded tau has been shown to promote the formation of tunneling nanotubes [140], possibly as an attempt to clear the cytoplasm from toxic proteins that cannot be otherwise cleared. However, the formation of tunneling nanotubes—or cellular contacts in other forms—is not required for cell-to-cell transfer of tau oligomers and aggregates, as several studies have shown that tau pathology can be transferred from one cell to another via the extracellular medium without cell-to-cell contacts or presence of donor cells [118, 121].

### Vesicle-mediated mechanisms of tau secretion

Cellular protein secretion mechanisms are classified into conventional and unconventional. Conventional secretion mechanisms include the classical secretory route, also known as endoplasmic reticulum (ER)–Golgi pathway, for proteins that carry a signal peptide, which directs them to the ER membrane [141]. A vast majority of secreted proteins use the classical secretory route. It is estimated that 39% of the human protein-coding genes will produce one or more products with a signal peptide, and 15% will produce a conventionally secreted protein product (proteinatlas.org). Unconventional secretion covers all the other routes, from PM pore formation [unconventional protein secretion type I (UPS I)] to organelle hitchhiking and exosomal secretion [unconventional protein secretion type III (UPS III)] and Golgi bypass [unconventional protein secretion type IV (UPS IV)] [142]. Unconventional protein secretion pathways are in most cases induced by cellular stress and are thus an important cellular survival strategy under proteostatic stress.

Different secretion mechanisms involving vesicular structures have been suggested for tau. One mechanism is UPS III-like and relies on intracellular structures, such as multivesicular bodies or endo-lysosomes, and may result in the secretion of tau inside small extracellular vesicles (~ 40 to 120 nm in diameter) called exosomes, but also in release of free tau, unbound to vesicles, through the fusion of endo-lysosomes with PM. Another mechanism, called microvesicle shedding, does not involve intracellular vesicular structures. The tau-containing vesicles are formed directly by outward budding of the PM, resulting in the secretion of tau in larger extracellular vesicles (~ 100 to 1000 nm in diameter) called ectosomes.

Exosomes are generated in multivesicular bodies, a type of late endosomes, following inward budding of the outer endosomal membrane. Tau has been reported to be found in exosomes purified from brains of tau(P301L) transgenic mice [143, 144] and in exosomes isolated from neuroblastoma cells and from cerebrospinal fluid of AD patients [145]. Importantly, exosomes may deliver pathological tau to unaffected cells, seeding further tau pathology. Neuron-derived exosomes extracted from plasma of mild cognitive impairment (MCI) and AD patients are able to promote tau pathology in wild-type mice [146]. Finally, cell-to-cell transfer of tau via exosomes can be facilitated by microglia, whose role in prion-like propagation of protein aggregates remains incompletely understood [147].

Besides multivesicular bodies, tau may also be found in endo-lysosomes. Fusion of endo-lysosomes with the PM releases tau and other proteins to the extracellular space in a vesicle-free form [148]. Tau may be delivered to endo-lysosomes by multiple protein trafficking pathways. First, during cellular stress and overloading of the ubiquitin–proteasome clearing system, a misfolding-associated protein secretion pathway (MAPS) can be activated to translocate misfolded proteins, including tau, to endo-lysosomes for secretion to the extracellular space [148, 149]. The MAPS pathway involves the initial capture of misfolded proteins by the chaperone ubiquitin carboxyl-terminal hydrolase 19 (USP19) at the extracellular surface of ER. Then, co-chaperones Hsc70 and DNAJC5 mediate the sorting of misfolded cargos into the lumen of endo-lysosomes [148, 150]. Alternatively, dysfunctional cytoplasmic proteins and protein aggregates can be delivered to endo-lysosomes through macroautophagy [148]. Several reports have suggested a role of macroautophagy in tau secretion [151–154]. It is not clear, however, if autophagosomes deliver tau to endo-lysosomes for secretion or if they are capable of directly fusing to the PM for cargo release.

Ectosomes are larger than exosomes and are generated by outward budding of the PM rather than originating from intracellular multivesicular bodies [155]. Tau has been found in ectosomes purified from neuroblastoma cells, primary cortical neurons, mouse brain interstitial fluid (ISF) [155], and from cerebrospinal fluid of AD patients and healthy controls [156]. Interestingly, tau secretion in ectosomes was suggested to be a normal physiological phenomenon, while exosomal tau secretion may prevail under pathological conditions [155, 156].

It thus appears that tau utilizes different non-exclusive routes to overcome cellular membrane barriers and to exit the cell. It seems plausible that under proteostatic stress, the cells would activate multiple unconventional secretion pathways to clear the cytoplasm from the misfolded and aggregated proteins which endanger cellular survival. It is not clear yet whether different conformational tau species have preferred release pathways, and whether the pathogenicity of different conformers depends on them.

It is important to note that many of the studies that have reported secretion of tau in exosomes or ectosomes also demonstrated that a significant fraction of extracellular tau appears to be in the vesicle-free form. Additionally, some studies have described the secretion mechanisms utilized by tau independent of vesicular secretion (Table 2 and discussed below). The high relative level of vesicle-free tau compared to tau found in extracellular vesicles could be explained by three scenarios: (1) tau may be secreted in a free, non-vesicle-associated form, with a minority secreted in vesicles, (2) tau may be secreted exclusively in vesicles, but rapidly escaping most of the vesicles, or (3) tau may be secreted exclusively in free form, but may associate with extracellular vesicles after secretion. While most likely these scenarios are not mutually exclusive, increasing body of evidence suggests that tau release to the extracellular space may occur via unconventional secretion directly through the PM.

### Tau secretion directly through the plasma membrane

Several studies suggest that while a fraction of the total extracellular tau is indeed found in exosomes or ectosomes, a large fraction of the extracellular tau pool exists in vesicle-free form [149, 155, 157, 158] (Table 2). Two recent studies demonstrated that tau secretion can occur via a type I mechanism of unconventional protein secretion (UPS I), i.e., by direct translocation across the PM [67, 159]. Using complementary methodological approaches, both groups concluded that direct secretion of tau through the PM involves the following key steps: (1) recruitment and clustering of tau at the cytosolic leaflet of the PM involving hyperphosphorylation of tau and specific lipids such as phosphatidyl inositol 4,5 phosphate (PI(4,5)P_2_) cholesterol and sphingolipids, and (2) release from the PM facilitated by binding to heparan sulfate proteoglycans (HSPG) located at the extracellular leaflet of the PM (Fig. 2). Although neither paper presented direct evidence of membrane pore formation, Katsinelos and colleagues showed that tau was capable of disrupting large unilamellar vesicle (LUV) membranes in a PI(4,5)P_2_-dependent manner [67]. Interestingly, many of these mechanistic aspects of tau secretion have significant similarity to the unconventional secretion of fibroblast growth factor 2 (FGF2), one of the most widely studied unconventionally secreted proteins [160].

Tau localizes to the PM in a clustered manner, and this localization is enhanced by overexpression or hyperphosphorylation, possibly as a consequence of increased levels of cytosolic tau or enhanced aggregation. Transmission electron microscopy revealed two types of tau clusters, with diameter ranging between 50 and 200 nm, at or very close to the PM [159]. Similar, reversible cation-sensitive clustering of tau has been previously reported for recombinant human tau in supported brain lipid membranes [161]. Experiments with large unilamellar vesicles (LUVs) demonstrated that tau and particularly its phosphomimetic mutant or hyperphosphorylated forms are able to bind to PI(4,5)P_2_ and this interaction may be required for membrane binding of tau [67]. Several groups have shown that phosphorylation promotes tau secretion through the PM [66, 67], although one study correlates tau recruitment to the PM with hypophosphorylated states [162]. While FGF2 secretion requires specific phosphorylation by the Tec kinase at Y82, tau secretion seems to depend on the general level of phosphorylation rather than phosphorylation of a specific amino acid residue [67, 163, 164]. Plasma membrane localization and secretion of phosphomimetic mutant of tau were enhanced compared to wild-type tau [67]. Furthermore, the phospho-specific tau antibodies AT8 (Ser202/Thr205) and PHF13 (Ser396) stained mostly tau at or near the PM in neuroblastoma cells overexpressing tau [159]. Other post-translational modifications may also be involved in the secretion process. The two cysteine residues, located in the R2 and R3, may play a role in tau secretion as suggested by findings that at least some secreted tau species are disulfide bridged [165]. FGF2 secretion also involves intermolecular disulfide bridge formation, which may serve as a driver for FGF2 oligomerization at the membrane [166]. While there is no known mechanistic or pathophysiologic link between tau and FGF2, the similarities in their secretion mechanisms raises the possibility that tau could obtain a specific conformational state which may be able to engage an FGF2-like unconventional protein secretion mechanism at the PM. More broadly, this highlights the importance of UPS in understanding how pathological proteins may be released from cells.

To escape cells directly through the PM (or other membranes), tau would have to be able to interact with the membrane lipids. Indeed, tau interacts with both biological [13, 162, 167–170] and artificial membranes [17, 50, 171–175]. Binding of tau to membranes in vitro is enhanced by the presence of anionic lipids such as phosphatidyl serine (PS) and PI(4,5)P_2_ [17, 67, 172, 175]. In a cellular context, tau may also require the presence of cholesterol and sphingomyelin for membrane interaction and penetration, indicating that cholesterol and sphingomyelin-dependent membrane microdomains (also called “lipid rafts”) may play a role in this process [18, 159, 176]. Interestingly, tau may also disrupt ordered membrane domains and may thus affect the function of the PM more broadly [173].

Binding of tau to membranes has several important, related consequences that may affect its physiological and pathological functions and its secretion process. Firstly, tau can deform membranes in vitro. Tau can induce micellarization of anionic fatty acids [177] and deform micelles [17]. When tau was incubated with vesicles, tau–lipid complex formation resulted in morphological deformation of the vesicles [175]. Importantly, while individual MTBDs of tau can induce vesicle lysis [174]), full-length tau does not seem to share this activity, so tau membrane interaction does not seem to cause cell death per se [172]. To date, however, no biological consequences for this membrane deformation have been directly shown. Second, tau clusters [159, 161] and fibrillizes upon binding to anionic lipid membranes [49, 172, 177]. Tau fibrillization requires the presence of negatively charged nucleator, and anionic lipids in membranes can serve such a function. The inner leaflet of the PM of animal cells is composed of about 20% of anionic lipids that provide negative charges and create an electrostatic field for the inner leaflet. Thus, anionic membranes located at the inner leaflet of the PM can recruit tau to the PM and nucleate tau fibrillization. Third, tau, a natively unstructured protein acquires α-helical conformation upon binding to membranes. The MTBDs of tau, especially R2 and R3, have been shown to undergo context-dependent folding on lipid membranes [17, 171, 175, 178]. The forming α-helices are amphipathic, and mutating key hydrophobic residues in the helices inhibit binding of tau to membranes [175] and may therefore be important in tau secretion. These helices do not penetrate deeply into the lipid bilayer, but could act as molecular tweezers to extract negatively charged phospholipids from the membrane [17, 175]. Similar short α-helices are also formed upon tau binding to microtubules [179], indicating that context-dependent folding of tau may take place with several interaction partners. Tau interaction with PM may also be partially mediated by interaction of tau N-terminus to PM-located proteins, such as annexin A2 and annexin A6 [180]. While membrane localization, deformation and membrane-dependent folding (and secretion) of tau could be important for the pathological forms of tau, it remains unclear if the lipid and membrane interactions play a role in physiological functions of tau in healthy cells.

A critical step in FGF2 secretion is the formation of a membrane pore by membrane-penetrating FGF2 oligomers. Tau was shown to disrupt membranes by forming pore-like amyloid structures [181, 182]. Importantly, post-translational modifications and pathological mutations may enhance this property [181, 182]. Tau aggregation/oligomerization inhibitors significantly suppressed secretion of human tau overexpressed in cultured cells as well as endogenous tau from murine primary neurons [159]. While direct evidence of formation of membrane pores during the secretion process is lacking, these findings suggest that tau could penetrate the PM through an oligomerization‐mediated process, somewhat similarly to FGF2. Interestingly, other disease-associated proteins, such as α-synuclein and Aβ, also have the ability to form bacteriotoxin-like annular protofibrils and pore-like structures in membranes [183, 184], suggesting that this mechanism could play a central role in unconventional secretion of several misfolded aggregating proteins capable of transcellular propagation. Importantly, membrane disruption by protofibrils could also provide an escape route from vesicles, both during the secretion process and after cellular uptake.

Lastly, like FGF2, tau secretion was also shown to depend on HSPG on cell surface to complete the translocation across the PM [67, 159]. Tau has long been known to bind to GAGs in vitro, and heparin is widely used as a promoter of tau aggregation [185, 186]. Tau secretion was efficiently suppressed when the cellular level of glycosaminoglycans (GAGs) was decreased by treatment with either sodium chlorate, a compound that inhibits sulfation of newly synthesized GAGs, or with heparinases, enzymes that specifically cleave heparin and heparan sulfate-type sulfated glycans [67, 159]. Chondroitinase ABC did not alter tau secretion indicating that HSPGs, but not chondroitin sulfate proteoglycans, are involved in the tau secretion process. Furthermore, tau secretion was impaired in CHO_745_ cells, which are deficient in the synthesis of proteoglycans due to lack of xylosyl transferase activity, but could be returned to almost normal level when wild-type cells were added to the culture, suggesting that even the presence of GAGs on neighboring cells is sufficient to facilitate tau secretion [67]. Importantly, sodium chlorate was able to reduce the secretion of endogenous tau from primary neurons [67, 159] suggesting that physiological secretion of tau may also involve GAGs. Altogether, these data suggest an important role for cell surface proteoglycans, specifically HSPGs, in tau secretion.

### Cellular uptake and templated misfolding of tau in recipient cells

After being secreted to the extracellular space, pathological tau oligomers, monomers or aggregates need to enter another cell via a process called cellular uptake. Following cellular uptake, pathological tau seeds may be degraded, re-secreted or can mediate the misfolding of healthy tau molecules in the recipient cells (Fig. 2). As for secretion, diverse cellular mechanisms can mediate tau internalization from the extracellular space. Recipient cells seem to favor low molecular weight, short tau fibrils over tau monomers and larger filaments [187], which may at least partially determine the potency of various tau aggregate species for cellular propagation [188]. Importantly, uptake of tau aggregates is not specific for neurons, and cell-to-cell transfer is likely to occur also between neurons and glial cells. Close proximity of the PMs of the donor and recipient cells, as is the case in synapses, appears to facilitate cell-to-cell transfer of tau aggregates [118].

Tau oligomers can be internalized by dynamin-dependent bulk endocytosis and transported within recipient cells in the endo-lysosomal pathway [187]. Micropinocytosis, another type of bulk and non-specific endocytosis, seems to play a central role in pathological tau uptake both in vitro and in vivo [189, 190], as well as for other pathologically misfolded proteins such as α-synuclein, TDP-43 [191], and PrP [192]. Before being internalized by micropinocytosis, tau binds to HSPGs on the PM, which promote the rearrangements of the membrane prior to endocytosis [189]. Binding of tau to HSPGs appears to be crucial for internalization to occur, and 6-*O*-sulfation pattern of the heparan sulfate sidechains was reported to be a critical determinant for tau binding [193]. Interestingly, heparin-like GAG mimetics can mask the HSPG binding site on tau, resulting in reduced cell surface binding, uptake and seeding of tau oligomers [189].

HSPG-dependent macropinocytosis is initiated by small protein aggregates, and tau trimers were shown to be the minimum size to initiate this mechanism [193, 194]. In line with this, a recent study demonstrated that different tau species can be internalized by different cellular mechanisms [195]. Macropinocytosis is the preferred entry route for tau monomers and small oligomers (although they can also be internalized by endocytosis), while dynamin-dependent endocytosis is favoured for bigger aggregates.

Macropinocytosis and HSPGs may also be responsible for the uptake of whole exosomes [196], although it is possible that exosomes are internalized as a result of the unspecificity of micropinocytosis. In general, endocytosis and/or pinocytosis has been reported to be a favored internalization route for exosomes over direct fusion to the PM [197, 198].

Specific interactions with cell surface receptors may be involved in internalization and toxicity of tau oligomers. For example, tau interacts with muscarinic receptors, which may mediate tau internalization via clathrin-mediated endocytosis and contribute to tau toxicity by disrupting calcium homeostasis [199]. Blockage of muscarinic receptors indeed attenuates tau-mediated neurotoxicity in vitro [20].

Once tau is internalized from the extracellular space into the endosomes, it must find a way to escape the endosomal vesicles to interact with and template misfolding of cytosolic healthy tau molecules and to fully propagate the pathology to the recipient cells. Tau is indeed capable of rupturing the endosome membrane [200, 201], although the molecular mechanisms of this process, or which forms of tau mediate it, have not been fully elucidated. Organized tau conformations such as annular protofibrils and pore-forming structures [181, 182] could be involved in mediating tau escape from endo-lysosomal vesicles by disrupting the membrane in a manner recalling the escape of viruses or bacteria from endosomes [202]. Interestingly, C-terminal fragments of tau-containing MTBDs may be particularly effective in lysing vesicles [172, 174].

The concept of transcellular propagation of misfolding pathology implies that a pathological protein aggregate originating from a single or a restricted group of cells is able to promote pathology broadly to different brain areas in a way that the further misfolded and aggregated molecules resemble in morphology and pathogenicity of the original seed. Accumulating evidence demonstrates the ability of pathological tau to be internalized and to drive the conversion of healthy tau molecules into pathological aggregates, both in vitro and in vivo [112, 118, 133, 188]. Moreover, the morphological features of the original tau seeds can be faithfully recapitulated in the newly formed tau aggregates [74, 135]. Moreover, specific monomeric conformers have been shown to have different intrinsic abilities to become pathogenic, and they can give rise to a limited amount of strains, suggesting that the misfolding is closely related to the structure of the original seed [40, 41].

Although mechanistic data are slowly emerging, it still remains unclear how the templated misfolding and seeding occurs at the molecular level. Two models have been proposed to explain the template seeding of endogenous tau. In the first model, called template-assisted growth, pathological oligomers act as templates for unfolded monomers, which are progressively packed into β-sheet structures and stabilized by hydrogen bonds [203, 204]. This model could explain the rapid process of tau fibrillization in vitro. In the second model, shared with PrP and Aβ [205] and called oligomer-nucleated conformational induction, monomers are not directly assembled into bigger aggregates, but they oligomerize before being incorporated into fibrils [206]. Unfolded tau monomers are stabilized to the highest energy state to constitute the seed of rearranged proteins, which then accelerates the assembly process [206, 207].

However, the pathway of tau fibrillization may not be as straightforward as it seems. A recent study demonstrated that different stable monomeric forms of tau with different seeding abilities coexist, implying that different seeds could originate from their aggregation [40]. This study also raises the possibility that tau, despite being natively unfolded, may physiologically exist in relatively stable conformations that affect its propensity to adopt a pathological form. Additionally, the complex pattern of tau post-translational modifications may also contribute to generation of different conformers. Different tau pools might have slightly different aggregation properties leading to formation of different pathological aggregates of the same protein, with distinct structural composition, shape, toxicity and ultimately different disease specificity [74].

It is also important to note that, similarly to other disease-associated low complexity proteins [208–210], tau can undergo liquid–liquid phase separation, where tau separates into liquid-like droplets similarly as in a water–oil demixing event [58, 211]. This process is mediated by the MTBDs and by electrostatic interactions between the negatively charged N-terminus and positively charged C-terminus [58, 212]. Liquid–liquid phase separation spatially concentrates tau molecules, possibly favoring tau homo-oligomerization and aggregation. The concept that liquid–liquid phase separation may be transformed into permanent aggregation is now validated for several RNA-binding proteins associated with different neuropathological conditions [213]. Interestingly, TIA-1, an RNA-binding protein centrally involved in the formation of stress granules [214], has been shown to directly modulate tau pathology in vitro and in vivo [215, 216]. Interestingly, TIA-1-dependent recruitment of internalized tau oligomers to stress granules has been suggested to provide a hub for tau toxicity and seeding in recipient cells [190].

### Further considerations for understanding propagation of tau pathology in brain tissue

Microglia play important and diverse roles in neurodegenerative diseases. Microglia have beneficial functions, such as clearing cellular debris, protein aggregates and dying cells, but can also contribute to disease pathogenesis by, e.g., abnormal synapse elimination and chronic pro-inflammatory cytokine secretion [217]. Post-mortem studies with patients affected by different tauopathies revealed deposits of aggregated tau in reactive microglia [218]. As microglia do not normally express tau, this indicates that microglia had engulfed tau aggregates from the extracellular space, or as a part of engulfed debris from dead neurons. It has been shown that microglia have the ability to internalize tau to degrade it [219–221]. However, when the amount of aggregating protein surpasses the degradation capabilities of microglia, toxic tau aggregates appear in the cytoplasm and promote microglial dysfunction [222]. While a correlation between microglia activation and spreading of tau pathology has been reported [223], the actual role of microglia in actively transferring pathological tau to neurons, and importantly also which forms of pathological tau are transferred, has been unclear. Importantly, microglia are able to promote cell-to-cell spreading of pathological tau by secreting it themselves [147]. In this study, microglia internalized neuronally secreted tau from the extracellular space and packed it in exosomes—which appear to contain the most toxic forms of tau [127, 155]. Consistently, when exosome formation was inhibited, tau propagation in neurons was reduced both in vitro and in vivo. Furthermore, depletion of microglia strongly suppressed propagation of tau pathology in human tau(P301S) expressing transgenic mice [147]. Additionally, astrocytes are also able to internalize both fibrillar and monomeric tau, implicating a possible role for other glial cells in the process of spreading the pathology [224, 225].

Factors affecting tau secretion or uptake, or efficiency of clearance of tau seeds from the brain interstitial fluid and CSF, likely affect the rate of disease progression in tauopathies. Genetic variants are known to contribute to the CSF levels of tau [226, 227]. However, currently it is not clear if and how genetic factors modulate individual susceptibility and rate of disease progression by altering propagation of tau pathology in the brain. Some mechanistic connections have been reported between late-onset AD risk gene function and tau secretion [157]. Moreover, expression and activities of several enzymes participating in generation and modification of sulfated glycosaminoglycans, particularly heparan sulfate sulfotransferases, have been reported to be altered in AD brain and to affect accumulation of phosphorylated tau species [228, 229]. While altered levels and function of HSPGs may relate to many aspects of AD pathogenesis, including ApoE function and growth factor activity in the brain, it is notable that HSPGs appear to play a key role in both secretion and uptake of pathological tau species.

One important contributor to clearance of tau aggregates from the brain is the brain glymphatic system, a brain-wide interstitial solute clearance system composed of astrocytes, paravascular routes and dural lymphatics [230, 231]. Clearance of extracellular tau from the brain was significantly impaired in mice lacking functional central nervous system lymphatic system [232]. Importantly, the glymphatic system function can be adversely affected by stroke, traumatic brain injury or other comorbidities, such as type 2 diabetes, which could enhance spreading of tau pathology in the brain [233, 234]. Similar to brain levels of Aβ, sleep–wake cycle regulates brain ISF and CSF levels of tau in humans [235]. In P301S transgenic mice, increased wakefulness and sleep disruption increased ISF and CSF tau levels, tau aggregation and spreading over longer periods of time [236], suggesting that alterations in tau clearance can have a strong impact on progression of tau pathology.

There appears to be an intriguing connection between neuronal activity and tau secretion. Increased neuronal activity stimulates tau release in cultured cortical neurons [119], in human tau-overexpressing transgenic mice in vivo [120] and in human iPSC neurons [121]. Moreover, enhanced neuronal activity accelerates development and spreading of tau pathology in the brains of rTg4510 mice [121]. These data implicate that not only the pathological forms of tau, but also physiological “healthy” forms of tau are secreted by neurons in a manner that is regulated by neuronal activity. Currently, it is unclear which of the mechanisms involved in secretion of pathological forms of tau are also utilized in neuronal release of non-pathological forms of tau. HSPGs appear to be at least partially involved also in secretion of endogenous non-pathological forms of tau in cultured rodent neurons [67, 159]. Whether the neuronally secreted tau species are pathological and can seed tau pathology in neighboring cells could simply be determined by their conformational state. Somewhat unexpectedly, even tau monomers may carry such conformational information, as suggested by a recent study [41].

The findings that hyperexcitability promotes tau secretion may have important implications in the progression of tau pathology in AD patients. Aβ is known to promote hyperexcitability and subclinical epileptiform activity may contribute to cognitive decline in AD [237, 238]. It is possible that in regions vulnerable to early tauopathy, such as the hippocampus, amyloid pathology could trigger or promote the release and propagation of pathogenic tau seeds. In rTg4510 mice, optogenetic stimulation of the hippocampus leads to robust worsening of tau pathology [121], providing some experimental support for this type of mechanism.

Finally, all studies addressing tau secretion mechanisms should be performed thorough characterization of the secreted tau species, vesicular and non-vesicular, to improve comparability and interpretation of data from different sources. Another factor complicating comparison of different tau secretion studies is that some studies use full-length tau protein, while others use tau fragments, typically containing one or more MTBDs, yet the conclusions from most studies are generalized to tau (implicating the full-length tau protein). The diversity of tau arises from multiple levels, including splicing, conformational states, morphology of aggregates and filaments, and how this diversity is connected to transcellular propagation of tau is only beginning to be addressed.

## Concluding remarks

The presence of tau in the cerebrospinal fluid of AD patients was long considered a mere consequence of passive release from degenerating and dying neurons. Accumulating evidence over the last decade has convincingly shown that tau and other neurodegeneration-associated intracellular amyloid proteins, such as α-synuclein, TDP-43 and SOD1, can be actively secreted from cells and that these extracellular protein aggregates, or seeds, can propagate the misfolding pathology to healthy cells. It now seems that cell-to-cell transmission is a common mechanism for disease progression in most, if not all, neurodegenerative diseases.

Disruption of proteostasis can be particularly detrimental to neurons. Accumulation of misfolded aggregated proteins in cells triggers multiple cellular mechanisms that jointly aim to restore proteostasis and improve cell viability. Not surprisingly, pathological forms of tau can be secreted via multiple non-exclusive mechanisms, including both vesicular and non-vesicle-mediated pathways, some of which appear to be induced by accumulation of protein aggregates in cells. Also, unconventional protein secretion directly through the PM, similar to FGF2 secretion (UPS I-like), has recently been reported for tau. Many amyloid proteins have long been known to be membrane active, and formation of membrane pores may be involved in tau secretion but also in the escape of tau from vesicles, both in the extracellular space and after internalization to recipient cells. Although tau is considered a natively unfolded protein it appears to be capable of context-dependent folding, which could be important for both the aggregation process but also for the membrane interaction and secretion processes.

Tau aggregates can be diverse in terms of their morphology such as reflecting slightly different conformational states of the aggregated tau protein. It is currently unclear if particular secretion pathways are preferred by certain species of tau aggregates. It is noteworthy that recent studies suggest that even tau monomers may carry conformational information that can be replicated during the templated misfolding process, which can be propagated through generations of cells.

The fact that tau is physiologically secreted independently of aggregation raises the question if some forms of extracellular tau could have a yet undiscovered function in the nervous system. Whether physiological secretion of non-pathological forms of tau in neurons occurs via the same or overlapping mechanisms as the pathological forms of tau needs to be clarified in future studies. Importantly, both physiological and pathological secretion of tau appears to be connected to neuronal activity. The role of glial cells and the glymphatic system in the clearance and propagation of pathological protein aggregates deserves more attention in the future.

It is possible that cellular secretion and uptake mechanisms for the diverse range of disease-associated amyloid proteins are at least partially overlapping. Better understanding of the mechanisms of pathological protein propagation is expected to help develop novel therapeutic strategies for slowing down the progression of neurodegenerative diseases. For instance, the development of monoclonal antibodies or other compounds that specifically target, sequester or disassemble tau conformations involved in the propagation of pathology could slow down disease progression in tauopathies. Alternatively, inhibition of the processes that promote tau interaction with plasma membrane lipids or cell surface proteoglycans may decrease the extracellular pool of tau available for internalization by neighboring cells. Inhibition of the uptake of secreted tau species or interfering with the templated misfolding or stress granule association of tau in the recipient cells could provide another therapeutic strategy for halting transcellular propagation of tau pathology. However, mechanistically we should first aim at better understanding of the different roles and functions of physiological vs pathological forms of secreted tau and also clarify whether the same, overlapping and distinct secretion mechanisms are responsible for secretion of the two different pools of extracellular tau.

## Abbreviations

AD: Alzheimer’s disease
AGD: Argyrophylic grain disease
CBD: Corticobasal degeneration
CSF: Cerebrospinal fluid
EM: Electron microscopy
ER: Endoplasmic reticulum
FGF2: Fibroblast growth factor 2
FTD: Frontotemporal dementia
FTDP-17: Frontotemporal dementia with Parkinsonism in chromosome 17
GAG: Glycosaminoglycan
HSPG: Heparin sulfate proteoglycans
IDP: Intrinsically disordered protein
MAPS: Misfolding-associated protein secretion
MT: Microtubules
MTBD: Microtubule-binding repeat domain
MVB: Multivesicular body
NFT: Neurofibrillary tangles
PD: Parkinson’s disease
PiD: Pick’s disease
PI(4,5)P2: Phosphatidyl inositol 4,5 phosphate
PFF: Pre-formed fibrils
PM: Plasma membrane
PrP: Prion protein
PS: Phosphatidyl serine
PSP: Progressive supranuclear palsy
TBI: Traumatic brain injury
UPS: Unconventional protein secretion
